# Hospital and Health News

**Published:** 1924-08

**Authors:** 


### HOSPITAL AND HEALTH NEWS.

### Alexandra Day.

The collections in London on Alexandra Day amounted to £40,100.

### Willesden General Hospital.

Mr. R. J. Hearne has been appointed secretary of the Willesden General Hospital.

### A Substitute for Cocaine.

A famous German chemist, Professor Willstaetter, has produced a new drug, "Psikain," which is described as far superior to cocaine and free from its sometimes dangerous after-effects.

### Cabs for Out-Patents.

The yearly contribution of £500 to St. Bartholomew's Hospital for the conveyance of out-patients home by taxi-cab after minor operations enabled 445 separate journeys to be made. The average fare was 4s.

### Proposed New Leeds Hospital.

The Ministry of Pensions is stated to be considering a site of ten acres for a large hospital at Leeds, where patients from a number of smaller hospitals in the north-east area will be concentrated. The number contemplated is 350.

### Memorial Wing at Blackburn.

The foundation stone has been laid of the war memorial wing which will add ninety beds to the Blackburn and East Lancashire Royal Infirmary. The cost is expected to be £90,000.

### £20 for Expenses at Lourdes.

Southwark Guardians have allowed a woman convalescent £20 to cover her expenses to Lourdes, the Minister of Health having told them that his sanction was not necessary if the expenditure was reasonable.

### The New Torbay Hospital.

The money given toward the new Torbay Hospital has been increased by £1,000 from Sir Mortimer Singer, who has promised a further £2,000 on condition that £10,000 is raised within three months.

### Modern Amenities for Mental Patients.

A cinematograph and a motor charabanc have been purchased for the use of patients at Parkside Mental Hospital. In the latter they go for drives three times a week. The patients also have a canteen where they may make small purchases out of their own money.

### In Need of a Convalescent Home.

The Prince of Wales' Hospital, Cardiff, for orthopædic cases, is in need of a convalescent home to which patients could be sent as soon as possible after operation. The home should be somewhere between Cardiff and Swansea. The hospital has been thanked for its services to disabled pensioners.

### Messrs. H. K. Lewis's Catalogue.

Messrs. H. K. Lewis and Co., Ltd., of 28 Gower Place, have just issued a supplement to the catalogue of their comprehensive circulating library, so invaluable to all interested in scientific or medical matters. The catalogue, which costs 1s. net, has the advantage of being cross-indexed according to subjects and to authors.

### A Blind Man's Gift of an Eye Department.

The new out-patient's ophthalmic department, which Mr. T. Singlehurst has given to the Northampton General Hospital, has been formally handed over. The generous donor has been blind for some years. A site is being reserved for an in-patient ophthalmic department which, it is hoped, another benefactor will eventually provide.

### Guy's Bi-centenary and Debt.

On January 6, 1925, the bi-centenary of Guy's Hospital will be celebrated. The latest report records a debt of £150,000, which the governors hope will be met before that date. The interest on this debt, amounting to £6,000, equalled the voluntary payments of the patients in the wards, and the income for the year fell short of the expenditure by £8,000.

### Enham Village Centre.

With reference to the account of Enham Village Centre, which appeared in a recent issue, we are asked to point out that, of the sum of £4,000 given by Princess Mary and Lord Lascelles, £2,800 only was spent on the four Royal cottages, the remainder being placed to the Endowment Fund for helping to carry on Enham as a permanent employment settlement for the disabled.

### Rebuilding Monk Wearmouth Hospital.

The governors of Monk Wearmouth and Southwick Hospital have approved the plan to build a new general hospital in Newcastle Road on the site acquired in 1920 for this purpose. The new hospital will contain 160 beds, forty of which will be reserved for paying patients. The cost is expected to be £160,000.

### Royal Visit to Bethlem.

The Duke of Connaught, who became a governor of the Bethlem Royal Hospital in 1922, recently paid a private visit to the institution and unveiled a tablet in the surgical ward recording his visit. The ward has been named after him, thus continuing the tradition by which every part of the building is called after some past or present member of the Royal Family.

### Middlesex Hospital Medical School.

Mr. S. A. Courtauld, who has for some years been a generous supporter of the Middlesex Hospital and its medical school, has given the munificent donation of £20,000 to endow the University Professorship of Anatomy in the Medical School. It may be recalled that the sister Chair of Physiology was endowed at this Medical School by a gift of a similar amount from an anonymous donor some two years ago.

### Sanitary Inspectors' Association.

The Thirty-seventh Annual Conference of Sanitary Inspectors will be held at Whitley Bay from September 2nd to September 5th. The subjects for discussion include Housing, Humane Slaughtering of Animals, Food Orders in Relation to Milk. All particulars can be obtained from the Hon. Secretary of the Sanitary Inspectors' Association, 15 Bessborough Street, S.W. 1.

### Rescue and Preventive Institutions.

**A useful handbook has been published by the Public Health Committee of the London County Council giving information as to Rescue and Preventive Institutions, including Homes and Hostels for those suffering from venereal disease, and institutions dealing with cases of mental deficiency. Copies may be obtained from Messrs. P. S. King and Sons, of Great Smith Street, Westminster, at the price of 1s. 3d.**

### Extensions to Chelmsford and Essex Hospital.

Among the points of interest in the extensions to the Chelmsford and Essex Hospital, which have been opened free of debt, at a cost of £29,000, are the complete block for the domestic staff, and the rubber that covers the floors of the two new wards. The hospital was last extended in 1909, and the new men's ward has been named after Mr. Ernest Ridley, chairman of the Building Fund.

### Hospital for Miners.

The miners of Hemsworth and South Kirby collieries with money provided by the Miners' Welfare Scheme propose to build a hospital, for the two parishes, at a cost of £18,000. It will have thirty beds for surgical cases to start with, and the men of the two collieries have agreed to raise a levy, amounting to £3,000 a year, for its maintenance. The Hemsworth Rural Council proposes to sell a site and then to return the purchase-money as a donation.

### Jenny Lind and the Colman Family.

The surgery, erected as a war memorial, and the Laura E. Stuart Ward, provided by a bequest from Mrs. Stuart, have been dedicated and opened at the Jenny Lind Hospital, Norwich. Mrs. Stuart was a member of the Colman family, which has many links with the institution. The present vice-chairman, who is also Lady Mayoress, and the present Lord Mayor, Miss Ethel M. Colman, are both sisters of the late Mrs. Stuart, who joined the committee of management of the hospital in 1883.

### Meeting of Blind Masseurs.

The sixth annual general meeting of the Association of Certificated Blind Masseurs was held in the Armitage Hall of the National Institute for the Blind. The meeting was presided over by Sir Robert Jones, and the chair was taken by Mr. Michael Whitfield, who is himself a blind masseur. Sir Robert spoke of the excellent work done by members of this Association and expressed the desire that the Association would extend its sphere of usefulness over an even greater area.

### The Hippocratic Oath.

Mr. W. H. S. Jones has edited the earliest Greek, Latin and Arabic MSS. of the "Hippocratic Oath," the famous medical oath upon which the ethical rules of the profession have been based. In addition to the MSS., the volume will contain translations, an essay and an appendix; it will be entitled "The Doctor's Oath: an Essay in the History of Medicine," and will be published by the Cambridge University Press.

### Nuneaton's Sunshine Home.

The Nuneaton Health Committee has formed a Sunshine Home at Tuttle Hill for predisposed consumptive children. The committee, which claims to be the first local authority to start this treatment, is to extend its experiment. The Isolation Hospital was taken over for the purpose, and cases which did not respond to the treatment at the clinics rapidly improved. Some twenty have been treated this year for an average period of two months. The age of the children is from five to twelve. There is no school, but a paddling pool, miniature sand beach, swings and innumerable games. The success and cheapness of the treatment will commend it to all local authorities.

### Savill Memorial Lecture.

On July 3, Dr. Arthur Hurst delivered the Savill (Memorial) Lecture of the West End Hospital for Nervous Diseases, at the Royal Society of Medicine. The subject was "Migraine." Sir William Hale-White, President of the Royal Society of Medicine, presided and after the lecture presented the Savill Prize and Medal to the successful candidate, Dr. M. E. Franklin, by proxy. This prize is awarded annually for the best thesis written by any student who had attended the Hospital for instruction. The lecture and prize were founded in memory of the late Dr. Thomas Dixon Savill for the promotion and encouragement of research and education in Neurology.

### Research in Anæsthetics.

Dr. Waldo, the City and Southwark Coroner, has twice recently returned a verdict of "Death by misadventure" at inquests on a man and child who had died after being operated upon at Guy's Hospital. Last year he had eleven cases in which death was accelerated by the administration of anæsthetics, notably chloroform, which was six times more dangerous than ether. Research was, he said, urgently needed in connection with anæsthetics in order to supplement the inquiry carried out in the coroner's court, and could best be done by the appointment of a small standing scientific committee nominated by the Home Office and paid out of public funds. He is also of the opinion that whole-time anæsthetists might usefully be appointed in all large general hospitals.

### Royal Commission on Lunacy Law.

The King has been pleased to approve the appointment of a Royal Commission to inquire as regards England and Wales into the existing law and administrative machinery in connection with the certification, detention, and care of persons who are or are alleged to be of unsound mind; and to consider the extent to which provision is or should be made for the treatment without certification of persons suffering from mental disorder. The Chairman of the Commission is the Rt. Hon. H. P. Macmillan, K.C., and Sir Humphry Rolleston and Sir David Drummond are among those serving on it. Mr. P. Barter, of the Ministry of Health, is Secretary.

### An Ambitious Programme Completed.

The ambitious programme undertaken on the 150th anniversary of Leicester Royal Infirmary has been completed. The institution has now 400 beds, in place of 300, and this addition absorbs the new wing of three floors that has been added. A residence for doctors, the addition of sixty-six beds to the Nurses' Home, an enlarged laundry and out-patient department comprise the extensions. Of the £100,000 appealed for £20,000 remains to be raised. The income continues to grow and is now not far behind the expenditure. Sir George Blacker, Dean of the Medical School of University College Hospital, is reported to have said that the best

**hospital wards in the country are to be found in the new extension of the infirmary.**

### St. Mary Abbot's Hospital.

St. Mary Abbot's Hospital, Kensington, has a strong hold on the affection of nurses who have been trained and have worked there, and the summer reunion which took place in beautiful weather was well attended. The proceedings began with a service in the chapel, which with its many pictures, its altar lights and sanctuary lamp is a model of what such a building should be. A brief address was given by Mr. Lombardini, the chaplain, to whom the hospital owes so much, and Rubenstein's "The Angel" was sung by the Nurses Choral Society. Sir Arthur Stanley then presented medals to sixteen nurses, and tea was afterwards served in the garden.

### Conference of Public Health Workers.

A conference was held recently at Oxford of county public health authorities and voluntary organisations with the object of forming a standing joint committee to secure increased co-ordination in public health work in the county. Colonel W. H. Ashhurst presided. He said that, in his opinion, such a committee would be able to promote increased co-ordination between voluntary organisations and so prevent duplication and over-lapping; to receive suggestions and proposals from individual organisations; and to undertake responsible propaganda on health questions and to assist in the creation of a well-informed public opinion. The proposal was agreed to and Colonel Ashhurst undertook to communicate with the local authorities and organisations with a view to the election of representatives to serve on the Joint Committee.

### New Site for Ear, Nose, and Throat Hospital.

Negotiations have now been completed for the purchase of a site on which to rebuild the Royal Ear Hospital. This site is situated on the corner of Huntley-street, and Pancras-street, Bloomsbury, W.C., and has the great advantage of being adjacent to University College Hospital and the New Rockefeller Buildings. It is proposed to make this new hospital the most up-to-date ear, nose and throat hospital in the world, and to equip it with all the most modern facilities for research work to combat deafness. The chairman, Mr. Geoffrey E. Duveen, himself a victim of deafness, made it possible to commence this scheme by a gift of £15,000 to the hospital as a memorial to his late father, Mr. Henry J. Duveen, one of the founders of the famous art firm of Duveen Bros., of London, Paris and New York, who died in 1919.

### Annual Report of Queen Victoria Jubilee Institute.

The Queen Victoria Jubilee Institute for Nurses has recently issued its 32nd annual report. The general account shows an excess of payments over ordinary expenditure amounting to £3,956 17s. 8d. The sum of £5,935 14s. 8d. was received from those Approved Societies who are paying for the nursing of their members under the schemes arranged by the Institute, and £8,938 9s. 1d. has been provided by the Queen's Fund. Although the Council is glad to record good progress in all its branches, it is felt that steps should be taken to make the work of the Institute better known with a view to securing wider support, and with this end in view a Publicity Committee has been formed. Mrs. Frank Stobart has kindly consented to become hon. treasurer. Deep regret is expressed at the death of Miss Amy Hughes, one of the first Queen's nurses, and afterwards general superintendent and a member of the Council.

### Death of Mr. R. A. Owthwaite.

We much regret to record the death of Mr. R. A. Owthwaite, who was for forty-one years secretary of the City of London Maternity Hospital, City Road, from which position he retired in 1912. In conjunction with the late Dr. Heath Strange he founded the Hampstead General Hospital in 1884, and for many years was hon. secretary. From the formation of the Metropolitan Hospital Sunday Fund in 1872 he acted as its assistant secretary until 1907. In this connection it may be mentioned that for over forty years he never missed attendance at the three services held in St. Paul's Cathedral on Hospital Sunday and taking charge of the collections made. In association with the late Sir Henry Burdett he assisted in organising the Home Hospitals Association, and for some time was its hon. secretary. He also took an active part in the work of the British Hospitals Association, of which he was for several years hon. treasurer. Mr. Owthwaite was always closely associated with church affairs.

